# Quantitative late gadolinium enhancement cardiac magnetic resonance analysis of the relationship between ablation parameter and left atrial tissue lesion following pulmonary vein isolation

**DOI:** 10.3389/fcvm.2022.1030290

**Published:** 2023-01-09

**Authors:** Qian Wang, Bingyu Huang, Shengqi Huo, Junyi Guo, Haojie Li, Tao Jiang, Dewei Peng, Lintong Men, Dazhong Tang, Chunlin Xiang, Yi Luo, Xiu Pi, Lulu Peng, Yue Jiang, Mengying Zhu, Wei Shi, Sheng Li, Jiagao Lv, Li Lin

**Affiliations:** ^1^Division of Cardiology, Department of Internal Medicine, Tongji Hospital, Tongji Medical College, Huazhong University of Science and Technology, Wuhan, China; ^2^Department of Radiology, Tongji Hospital, Tongji Medical College, Huazhong University of Science and Technology, Wuhan, China

**Keywords:** atrial fibrillation, radiofrequency ablation, pulmonary vein isolation, lesion size index, magnetic resonance imaging, late gadolinium

## Abstract

**Background:**

The impact of ablation parameters on acute tissue lesion formation after pulmonary vein isolation (PVI) has not been sufficiently evaluated in patients with atrial fibrillation. Radiofrequency ablation lesion can be visualized by late gadolinium enhancement cardiac magnetic resonance (LGE-CMR). We sought to quantitatively analyze the relationship between ablation parameter and tissue lesion following PVI at different segments of pulmonary vein (PV) using LGE-CMR.

**Methods:**

Twenty-one patients with atrial fibrillation who underwent PVI procedure were retrospectively enrolled. All patients underwent LGE-CMR examination within 3 days after radiofrequency ablation. Ablation parameters during PVI were documented, including lesion size index (LSI), force–time integral (FTI), power, contact force, temperature, and time of duration. The ablation point was projected onto 3-dimensional (3D) left atrial shell constructed base on LGE-CMR and corresponding image intensity ratio (IIR) was calculated on the same shell. A tissue lesion point was defined when the LGE-CMR IIR was > 1.2.

**Results:**

In total, 1,759 ablation points were analyzed. The ablation parameters and IIRs for each PV segment were significantly different (*P* < 0.0001). IIRs corresponding to ablation points at posterior of PV tended to be higher than those at non-posterior of PV when similar ablation parameters were applied during ablation. LSI was a better predictor of tissue lesion existence following PVI than FTI, contact force, power, temperature, and duration time at non-posterior wall of PV. The IIR showed positive correlation with LSI at non-posterior wall of PV (non-posterior of right PV, *r* = 0.13, *P* = 0.001, non-posterior of left PV, *r* = 0.26, *P* < 0.0001).

**Conclusion:**

When similar ablation parameters were applied during PVI, the posterior wall of PV had more severe tissue lesion than non-posterior wall of PV. Therefore, it was reasonable to decrease ablation energy at posterior wall of PV. Moreover, LSI was a better index to reflect tissue lesion quality following PVI at non-posterior of PV.

## 1. Introduction

Radiofrequency catheter ablation is a widely practiced and effective treatment for atrial fibrillation (1). Pulmonary vein isolation (PVI) forms the cornerstone of atrial fibrillation ablation (2). Proper radiofrequency delivery during catheter ablation is crucial to obtain an effective lesion, whereas excessively high radiofrequency delivery can possibly lead to cardiac perforation, steam pop, and collateral damage (3, 4).

The left atrium is a thin-walled tissue in which radiofrequency ablation must be precisely titrated. It has been reported that the left atrium wall thickness is not uniform under the catheter ablation line, and excessive ablation for thin walls may induce potential damage to extracardiac structures (5). Furthermore, histological studies have revealed that the area surrounding the pulmonary veins (PVs) is anatomically heterogeneous (6). Based on these features, we hypothesized that similar ablation parameters applied during radiofrequency ablation may cause different tissue lesion at different location of left atrium.

Power, temperature, time of duration, and contact force are important determinants in ablation lesion formation and are usually monitored during radiofrequency ablation (7). However, these parameters provided limited accuracy in assessing lesion quality (8). Force–time integral (FTI) combines contact force and duration time to provide accuracy information on lesion size (9). However, FTI does not take the important role of power into account. Lesion size index (LSI) is a multi-parametric index that incorporates power, contact force, and duration time. *In vitro* studies have revealed that LSI provides better real-time information on ablation lesion size than FTI (10). Whether LSI could predict the extent of myocardial tissue lesion in human bodies has not been well investigated.

Late gadolinium enhancement cardiac magnetic resonance (LGE-CMR) is a non-invasive tool used to detect myocardial damage with high sensitivity (11). LGE-CMR imaging allows good visualization of acute ablation lesions (12). The image intensity ratio (IIR) was proposed to homogenize the CMR from different individuals, with an IIR > 1.2 representing unhealthy left atrial tissue intensity (13). We sought to quantitative evaluate the extent of left atrial tissue lesion using IIR.

The purpose of this study was to estimate the relationship between ablation parameter and tissue lesion following PVI at different segments of PV using LGE-CMR IIR.

## 2. Methods

### 2.1. Patients

**FIGURE 1:**
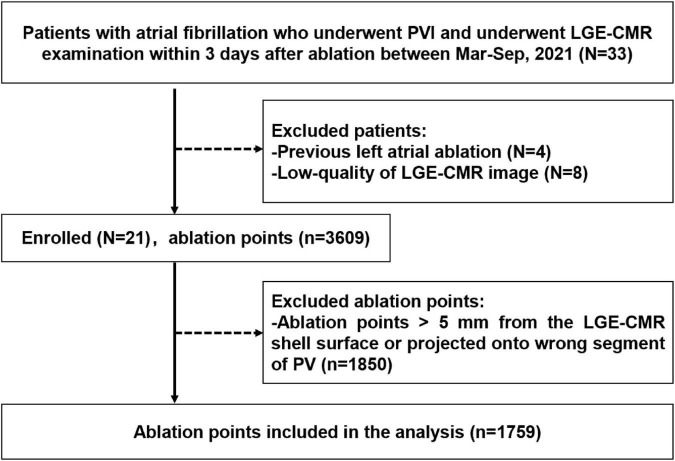
Flow chart of the study design and outcome. PVI, pulmonary vein isolation; LGE-CMR, late-gadolinium enhancement cardiac magnetic resonance; Mar, March; Sep, September; PV, pulmonary vein.

This was a single institution, retrospective, observational study. Participants were identified using the electronic medical record system of the hospital. A total of 536 patients with atrial fibrillation were identified underwent radiofrequency ablation procedure in our center during March 2021–October 2021. Among them 33 patients underwent LGE-CMR examination within 3 days after procedure. Four patients who had previous left atrial ablation and eight patients with inadequate LGE-CMR image quality were excluded. LGE-CMR image exclusion criteria included low quality image with defocus, image with artifacts passing through the left atrial region. Ultimately, 21 patients were included in the analyses. Patients who met the inclusion criteria were consecutively recruited. The flow chart of the study design was shown in Figure 1. Demographic characteristics were recorded from all cases. This study followed the Declaration of Helsinki, the research protocol was approved by the Institutional Ethics Committee of Tongji Hospital, Tongji Medical College, Huazhong University of Science and Technology (Approved ID:TJ-IRB20220814).

### 2.2. PVI procedure and AutoMark setting

Before the ablation procedure, transesophageal echocardiography was performed in all patients to exclude left atrial thrombus. Antiarrhythmic medications were discontinued at least five half-lives before the procedure. After achievement of femoral venous access, a 10-electrode catheter (Inquiry™, St. Jude Medical, MN, USA) was inserted and positioned into the coronary sinus, and a double *trans*-septal puncture was performed. Through *trans*-septal accesses, Swarts long sheath (St. Jude Medical, St. Paul, MN, USA) and Agilis steerable sheath (St. Jude Medical Inc., St. Paul, MN, USA) were placed into the left atrium. Heparin was administered to achieve and maintain activated clotting time values of 200–300 s throughout the radiofrequency ablation procedure. Under the guidance of EnSite Velocity TM V5.0 (St Jude Medical, St. Paul, MN, USA) 3-dimensional navigation and mapping system, 3D anatomical model of the left atrium and PV were constructed. Radiofrequency ablation procedure was performed under conscious sedation. Radiofrequency ablation was delivered using an open irrigated, fiber-optic CF-sensing ablation catheter (TactiCath™ Quartz catheter, TCQ, St. Jude Medical, MN, USA). The radiofrequency generator was set at temperature-controlled mode, with the temperature was limited to 43°C. The ablation power was 30 W in the posterior and inferior wall of left PV and 40 W in other areas of PV. The irrigation flow rate was 17–30 ml/min during ablation. The radiofrequency was terminated at each ablation point when the target LSI was reached at 4.0–4.5, and the catheter was moved to an adjacent point. The ablation was performed point by point until the PV potential was completely isolated. Locations and ablation parameters of ablation points were documented on 3D mappings using AutoMark system. This automated system recorded average power, temperature, contact force, duration time, and automatically calculated FTI and LSI when the ablation catheter stayed within the confined area.

### 2.3. LGE-CMR imaging acquisition and post-processing

All patients underwent LGE-CMR examination within 3 days after the PVI procedure using a 3-Tesla scanner system equipped with a 32 channel cardiac coil. At the time of LGE-CMR examination, all patients were in sinus rhythm. A total of 0.2 mmol/kg gadobutrol was injected 20 min before the examination. Axial projection was performed using a 3D ECG-triggered inversion recovery gradient echo sequence and free-breathing navigator. A total of 30–50 slices were set to obtain complete left atrium coverage.

**FIGURE 2:**
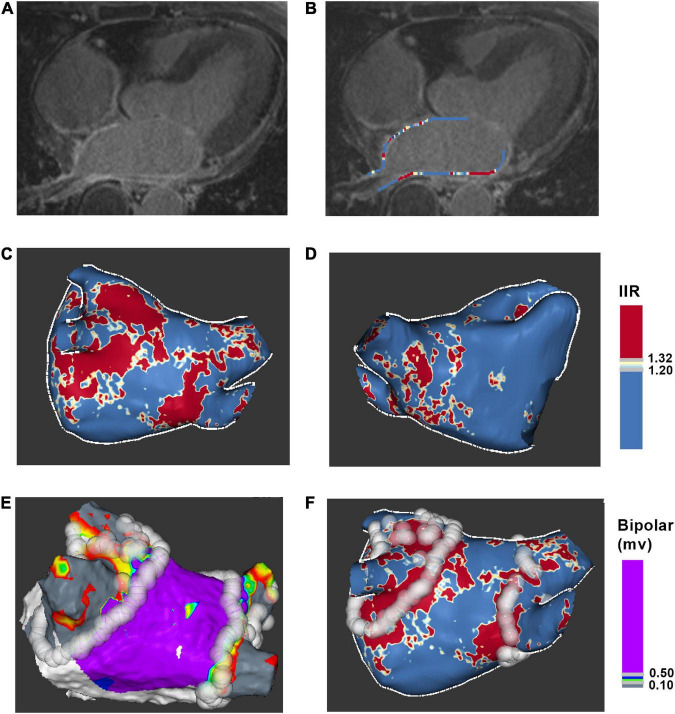
Process for ablation points projected onto LGE-CMR left atrial shell. **(A)** Acquire axial view of left atrium. **(B)** Manually drawn left atrial wall contour. **(C)** Posterior view of 3D color-coded LGE-CMR shell. **(D)** Anterior view of 3D color-coded LGE-CMR shell **(E)** 3D electroanatomic map of left atrium. **(F)** The merge of LGE-CMR shell and electroanatomic map. Ablation points were projected onto the surface of 3D LGE-CMR shell of left atrium. IIR, image intensity ratio.

Late gadolinium enhancement cardiac magnetic resonance images were post-processed using ADAS 3D LA software. Left atrial wall layer was manually drawn at each axial plane and was adjusted by the software automatically to build the 3D shell (Figures 2A, B). The IIR was calculated as each pixel signal intensity to the mean blood pool intensity ratio. IIRs were projected onto the 3D shell of left atrium and were color-coded (Figures 2C, D).

### 2.4. LGE-CMR imaging and electroanatomic map correlation

**FIGURE 3:**
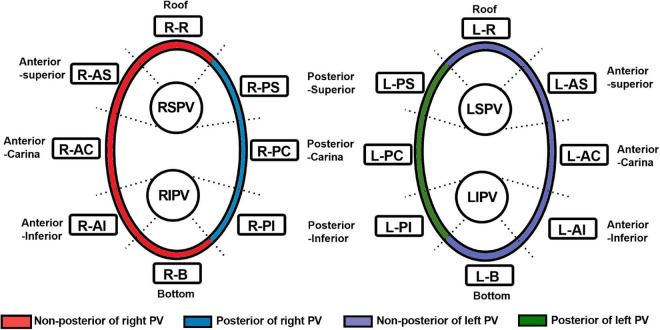
Segmentation of the pulmonary vein. RSPV, right superior pulmonary vein; RIPV, right inferior pulmonary vein; LSPV, left superior pulmonary vein; LIPV, left inferior pulmonary vein; R-R, right roof; R-AS, right anterior superior; R-AC, right anterior carina; R-AI, right anterior inferior; R-B, right bottom; R-PI, right posterior inferior; R-PC, right posterior carina; R-PS, right posterior superior; L-R, left roof; L-AS, left anterior superior; L-AC, left anterior carina; L-AI, left anterior inferior; L-B, left bottom; L-PI, left posterior inferior; L-PC, left posterior carina; L-PS, left posterior superior; PV, pulmonary vein.

Ablation points were projected onto the surface of 3D electroanatomic map (Figure 2E). Processed LGE-CMR left atrial shells were merged with the 3D electroanatomic maps using ADAS 3D LA software (Figure 2F). The fused model was refined using a series of characteristic fiducial points, for example, aortic root, mitral annulus, left ventricular apex, and left atrium, to further align the local mismatch of the two surfaces. Ablation points with projection distance > 5 mm further from the LGE-CMR shell surface or points projected onto wrong segment of PV were excluded from the analysis. Each PV antrum was divided into 8 segments under the ablation line, including roof, anterior-superior, anterior-carina, anterior-inferior, bottom, posterior-inferior, posterior-carina, and posterior-superior (Figure 3). The areas around the PVs were further divided into four anatomical regions, including non-posterior of right PV, posterior of right PV, non-posterior of left PV and posterior of left PV. The number of visual gaps in each segment was calculated. Visual gap was defined as a site > 4 mm with no gadolinium enhancement under the ablation line (14).

### 2.5. Statistical analysis

Statistical analysis of the data was performed using SPSS 25.0 software. Categorical variables were expressed as number (%), and data were compared using the χ^2^ test or Fisher’s exact test. For continuous variables, the normality test was firstly performed. Data were presented as the mean ± standard deviation (SD) or median (interquartile range, IQR) for continuous variables. The differences between two groups were analyzed using unpaired *t*-tests for normal distribution variables. The Mann–Whitney test were performed to compare non-normally distributed variables. The level of statistical significance was set at *P* < 0.05.

## 3. Results

### 3.1. Patient Characteristics

Between March 2021 and October 2021, 33 patients with atrial fibrillation received PVI ablation procedure and underwent LGE-CMR examination within 3 days after ablation. Four patients who had previously undergone left atrial ablation procedure were excluded. Eight patients were ruled out due to poor LGE-CMR image quality. Twenty-one patients who met the inclusion criteria were retrospectively included (Figure 1). Baseline characteristics of the patients were presented in Table 1. The average age of the patient population was 60 ± 5 years and 12 out of 21 (43%) were men. Sixteen patients had paroxysmal atrial fibrillation, and five patients had persistent atrial fibrillation. PVI was successfully performed in all patients. No major complications were observed during the ablation procedure.

**TABLE 1:** Baseline characteristics of the population (*N* = 21).

Demographics	Patients (*N* = 21)
Age (years), mean ± SD	59.7 ± 5.3
Men, *n* (%)	12 (57.1)
Hypertension, *n* (%)	9 (42.9)
Diabetes mellitus, *n* (%)	4 (19.0)
Hyperlipidemia, *n* (%)	3 (14.3)
Stroke, *n* (%)	1 (4.8)
CHA2DS2-VASC Score, mean ± SD	2.0 ± 1.2
AF duration (months), median (IQR)	5 (1–30)
Paroxysmal atrial fibrillation, *n* (%)	16 (76.2)
**Echocardiography data, mean ± SD**	
LA anteroposterior diameter (mm)	41.1 ± 5.0
LV anteroposterior diameter (mm)	49.3 ± 3.5
LV ejection fraction (%)	58.4 ± 5.7
**Underlying cardiomyopathies, *n* (%)**	
Mitral or aortic valve insufficiency	5 (23.8)
Hypertrophic	1 (4.8)
Heart failure	1 (4.8)
Time from PVI procedure to the MRI examination (days), median (IQR)	2 (2–3)

LA, left atrial; LV, left ventricular; SD, standard deviation, AF, atrial fibrillation.

### 3.2. Regional variations of IIR, ablation parameter, and visual gap

**FIGURE 4:**
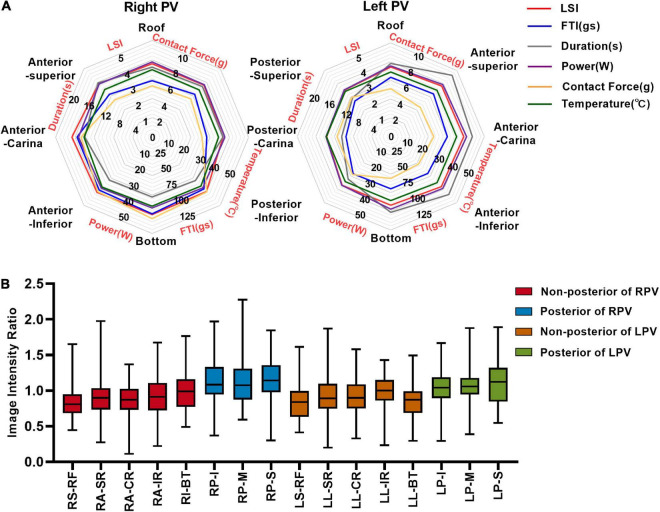
Image intensity ratio and ablation parameters of each PV segment under the ablation line. **(A)** The distribution of LSI, FTI, duration, power, contact force, and temperature at each segment of PV. **(B)** Myocardial wall image intensity ratio of each PV segment under ablation line. LSI, lesion size index; FTI, force–time integral; R-R, right roof; R-AS, right anterior superior; R-AC, right anterior carina; R-AI, right anterior inferior; R-B, right bottom; R-PI, right posterior inferior; R-PC, right posterior carina; R-PS, right posterior superior; L-R, left roof; L-AS, left anterior superior; L-AC, left anterior carina; L-AI, left anterior inferior; L-B, left bottom; L-PI, left posterior inferior; L-PC, left posterior carina; L-PS, left posterior superior; RPV, right pulmonary vein; LPV, left pulmonary vein.

**FIGURE 5:**
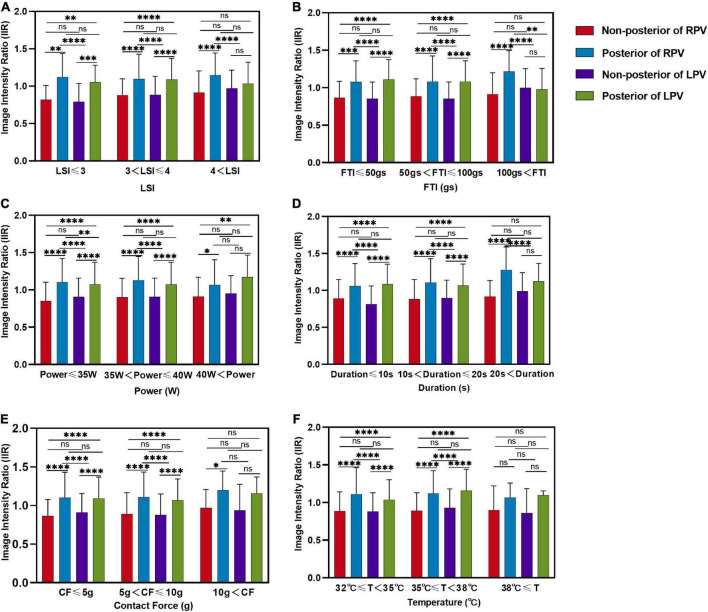
Comparison of image intensity ratios between different locations of left atrium under different level ablation parameter categories. Comparison of the image intensity ratio between non-posterior of right PV, posterior of right PV, non-posterior of left PV and posterior of left PV with three levels of **(A)** LSI, **(B)** FTI, **(C)** power, **(D)** duration, **(E)** contact force, **(F)** temperature applications. LSI, lesion size index; FTI, force–time integral; CF, contact force; T, temperature; ns, non-significant. *P < 0.05, ^**^P < 0.01, ^***^P < 0.001, and ^****^P < 0.0001.

A total of 1,759 ablation points were analyzed in this study. These ablation points were assigned to eight segments of PVs according to where they were projected on the left atrial shell. The ablation parameters applied at each PV segment showed large variation. Ablation parameters including LSI, FTI, power, temperature, and time of duration were lower at posterior wall (posterior-superior, posterior-carina, posterior-inferior) of left PV (Figure 4A and Table 2). The lowest contact force was observed at the left anterior inferior wall (left anterior carina, left anterior inferior, left bottom) of left atrium (Figure 4A and Table 2). The IIRs of the left atrial tissue corresponding to the ablation points were calculated using ADAS software. Figure 4B showed the distribution of IIR at each segment of PV. Posterior wall of PV had relatively higher IIR than non-posterior wall of PV. LSI applied during PVI were divided into 3 different levels. Compared with IIRs at posterior wall of PV, we found that IIRs at non-posterior wall of PV were higher when similar levels of LSI were applied (Figure 5A). Other ablation parameters were also divided into 3 different level groups, similarly, IIRs at posterior wall of PV tended to be higher than non-posterior wall of PV among these groups (Figures 5B–F). Visual gaps were detected in 12 (75%) PV segments (Supplementary Figure 1). A total of 16 (76.2%) patients showed at least one visual gap. The greatest number of visual gaps was six and were located in the anterior-carina of right PV. No gaps were detected in the posterior-carina, posterior-superior, bottom of left PV and posterior-carina of right PV. Furthermore, visual gaps concentrated in the anterior and roof walls [36 (81.8%)]. The incidence rate and distribution of gaps on LGE-CMR images were similar of those reported by other authors (15, 16).

**TABLE 2:** Ablation parameters for each segment under the ablation line.

Segment	*n*	LSI	FTI (gs)	Contact force (g)	Temperature (°C)	Duration (s)	Power (W)
R-R	147	3.9 (3.4–4.2)	73 (52–98)	5 (3–7)	35 (34–36)	13 (9–18)	39 (36–41)
R-AS	125	4 (3.65–4.2)	78 (49–102)	5 (3–7)	34 (34–36)	14 (10–20.5)	39 (36–41)
R-AC	121	4.1 (3.9–4.45)	95 (74–125)	7 (6–8)	35 (34–36)	13 (9.5–17.5)	38 (35–40)
R-AI	124	4.1 (3.8–4.4)	104 (71.5–123.8)	8 (7–9)	37 (35–37)	12 (9–16)	36 (34–40)
R-B	123	4 (3.7–4.3)	96 (73–121)	8 (6–10)	36 (35–37)	11 (9–15)	36 (34–40)
R-PI	81	4 (3.7–4.3)	88 (70–116.5)	7 (6–10.5)	36 (35–37)	12 (9–15)	37 (34–40)
R-PC	105	3.7 (3.4–4)	66 (49.5–91)	5 (4–6)	34 (34–35)	13 (10–18)	38 (35.5–40)
R-PS	100	3.8 (3.5–4.1)	70.5 (53.3–93.8)	5 (4.25–7)	34 (34–35)	13 (10–17)	38.5 (36–40)
L-R	99	3.8 (3.3–4.1)	75 (43–106)	5 (4–6)	34 (34–35)	14 (9–20)	38 (35–40)
L-AS	84	3.9 (3.5–4.2)	76.5 (47–107.8)	5 (3–6)	34 (34–36)	17.5 (12–23)	40 (37–42)
L-AC	125	4 (3.6–4.2)	73 (46.5–99.5)	4 (3–6)	35 (34–36)	15 (11–23)	40 (38–44)
L-AI	122	3.9 (3.5–4.1)	63 (44.5–91)	4 (3–5)	35 (34–36)	16 (12–22)	40 (36–42)
L-B	97	3.8 (3.2–4.1)	63 (35.5–97.5)	4 (3–6)	34 (34–35)	15 (11–19.5)	40 (37–40.5)
L-PI	84	3.7 (3.32–4.1)	65 (50.3–96.5)	6 (5–7)	34 (34–35)	11 (9–16)	38 (33.3–40)
L-PC	118	3.45 (3.2–3.7)	61 (47–72.3)	6 (5–7)	34 (33–35)	10 (8–12)	34 (32–36)
L-PS	104	3.5 (3.2–3.8)	65.5 (49.3–83)	6 (5–7)	34 (33–35)	11 (8.25–14)	35 (33–38)

LSI, lesion size index, FTI, force–time integral, R-R, right roof, R-AS, right anterior superior, R-AC, right anterior carina, R-AI, right anterior inferior, R-B, right bottom, R-PI, right posterior inferior, R-PC, right posterior carina, R-PS, right posterior superior, L-R, left roof, L-AS, left anterior superior, L-AC, left anterior carina, L-AI, left anterior inferior, L-B, left bottom, L-PI, left posterior inferior, L-PC, left posterior carina, L-PS, left posterior superior.

### 3.3. Prediction of tissue lesion existence after PVI at different anatomical locations of PV

**FIGURE 6:**
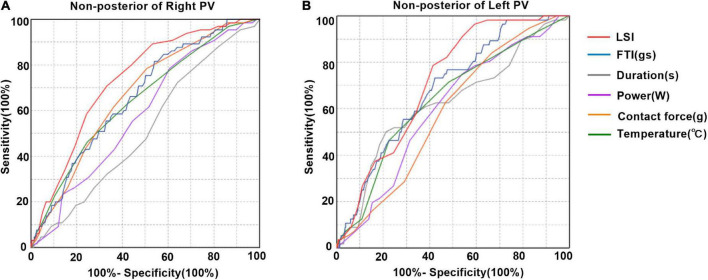
Prediction of tissue lesion existence after pulmonary vein isolation. **(A)** Receiver operating characteristic curve analysis for tissue damage existence predictability at non-posterior of right PV. LSI showed the best predictive value with an area under the Receiver operating characteristic (ROC) curve of 0.733 (95% CI 0.676–0.790). Area under the ROC curve for FTI, contact force, temperature, power and duration was 0.661, 0.665, 0.646, 0.582, and 0.514, respectively. The best cutoff value for LSI for tissue lesion was 4.15 (sensitivity 70.8%, specificity 66.8%). **(B)** Receiver operating characteristic curve analysis for tissue damage existence predictability at non-posterior of left PV. LSI showed the best predictive value with an area under the ROC curve of 0.703 (95% CI 0.647–0.759). Area under the ROC curve for FTI, contact force, temperature, power and duration was 0.668, 0.549, 0.635, 0.600, and 0.651, respectively. The best cutoff value for LSI for tissue lesion was 3.95 (sensitivity 78.6%, specificity 58.6%).

Tissue lesion point was defined when corresponding IIR was > 1.2. The LSI, FTI, power, temperature, and time of duration corresponding to the tissue lesion points were lower at posterior wall of left PV (Supplementary Figure 2). Receiver operating characteristic (ROC) curves were made for LSI, FTI, power, contact force, temperature, and time of duration to determine the parameter that best predict tissue lesion point existence. At non-posterior of right PV, LSI showed the best prediction value with an area under the ROC curve of 0.733 (95% CI 0.676–0.790) (Figure 6A). Similarly, LSI showed the best prediction value at non-posterior of left PV with an area under the ROC curve of 0.703 (95% CI 0.647–0.759) (Figure 6B). The LSI > 4.15 was highly predictive of tissue lesion existence at non-posterior of right PV (sensitivity 70.8%, specificity 66.8%). The LSI > 3.95 was highly predictive of tissue lesion existence at non-posterior of left PV (sensitivity 78.6%, specificity 58.6%). The areas under the ROC curves of these ablation parameters were close to 0.5 at posterior of PV, indicating that these ablation parameters had poor predictive performance of tissue lesion existence (Supplementary Figures 3A, B).

### 3.4. Relationship between IIR and LSI at different anatomical locations of PV

**FIGURE 7:**
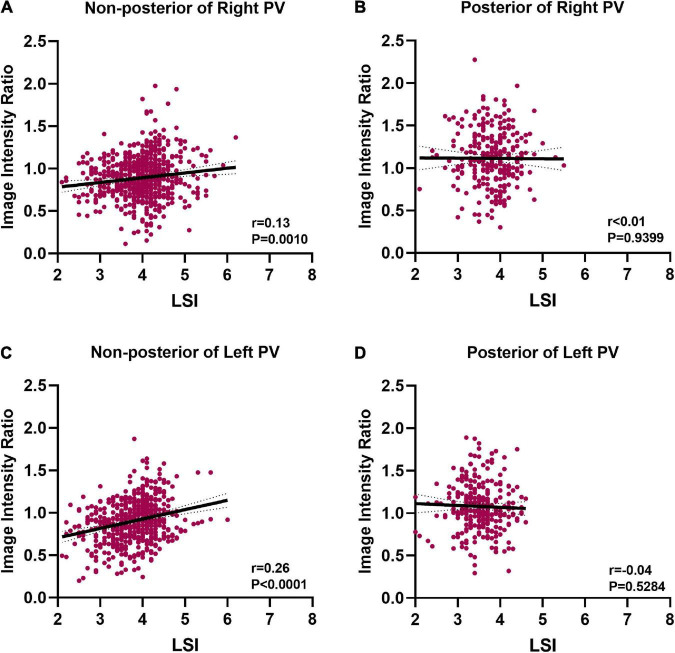
Correlation between image intensity ratio and LSI. **(A)** Positive correlation between image intensity ratio and LSI at non-posterior of right PV (*r* = 0.13). **(B)** There was no correlation between image intensity ratio and LSI at posterior of right PV. **(C)** Positive correlation between image intensity ratio and LSI at non-posterior of left PV (*r* = 0.26). **(D)** There was no correlation between image intensity ratio and LSI at posterior of left PV.

Positive correlations between IIR and LSI were found at non-posterior of right PV and non-posterior of left PV (*r* = 0.13 *P* = 0.001, *r* = 0.26 *P* < 0.0001, respectively) (Figures 7A, C). The IIR was not linearly correlated with LSI at posterior of right PV and posterior of left PV (*r* < 0.01 *P* = 0.9399, *r* = −0.04, *P* = 0.5284, respectively) (Figures 7B, D).

## 4. Discussion

This study described a new application of LGE-CMR IIR in evaluating the relationship between ablation parameter and left atrial tissue lesion following PVI. Our major findings were as follows: (1) Compared with non-posterior wall of PV, the posterior wall of PV had higher IIR when similar ablation parameters were applied during ablation. (2) ROC curve analysis identified that at non-posterior of PV, LSI was a better predictor of acute tissue lesion existence following PVI than FTI, contact force, average power, temperature, and duration time. (3) There were positive correlations between IIR and LSI at non-posterior wall of PV.

According to current atrial fibrillation ablation guidelines, lower radiofrequency energy applied on the posterior wall of the left atrium is recommended to reduce the risk of esophageal damage (2). The ablation procedures in this study were in line with the guidelines as lowest ablation parameters including LSI, FTI, power, temperature, and time of duration were observed at posterior of left PV. Consistent with previous studies that atrial ridge between ipsilateral PVs may reduce catheter stability (17, 18), we found contact force was lowest at left anterior inferior wall of PV. Several previous studies have reported that the thickness of the left atrial wall varies at different locations under the ablation line, with the posterior wall being thinner than the anterior wall of PV (5, 16, 19, 20). For thicker wall, the ablation parameters sufficient to create transmural lesions are higher. Conversely, excessive ablation for thin walls may bring damage to extracardiac structures. Furthermore, the composition of left atrial wall was not uniform at different anatomical site of left atrium, which may influence the conduction of radiofrequency heat to deep myocardial tissue (6). In this study, we found that IIRs tended to be higher at posterior wall of PV compared to the anterior wall of PV when similar ablation parameters were applied during PVI. Moreover, visual gaps concentrated in the non-posterior wall of PV. These results suggested that posterior wall of PV was more vulnerable to ablation injury. The ablation parameters applied at posterior wall of left PV were lower, while the corresponding IIR values were higher than non-posterior wall of PV. Therefore, the posterior wall of PV, especially the left posterior wall of PV, does not require excessive ablation during PVI. Firstly, it was easy to achieve adequate ablation efficacy in posterior wall of PV. Secondly, the extensive ablation energy delivered to the posterior wall of the left atrial might increase the incidence of atrial esophageal fistula.

The generation of myocardial lesion is the basis of successful atrial fibrillation ablation (21, 22). Power, temperature, duration time, and contact force are important parameters that determinate lesion quality (7). The FTI is an ablation index that combining contact force and radiofrequency time. Several studies have shown the correlation between FTI and lesion dimension during ablation *in vitro* (9). Recently, the LSI was developed to allow better prediction of lesion size. *In vitro* validations have shown that LSI was a better predictor of radiofrequency lesion dimension than contact force and FTI (10). The optimal LSI for creating appropriate ablation tissue lesion during PVI has not been verified. Our study found that LSI > 3.95 (sensitivity 78.6%, specificity 58.6%) was highly predictive of tissue lesion existence at non-posterior of left PV and LSI > 4.15 (sensitivity 70.8%, specificity 66.8%) showed relatively high sensitivity for predicting tissue lesion existence at non-posterior of right PV. The results indicated that LSI was a better predictor of tissue lesion existence following PVI than FTI, contact force, power, temperature, and duration time at the non-posterior wall of PV in patients with atrial fibrillation. However, ablation parameters had poor predictive performance for tissue lesion existence at posterior wall of PV. The posterior wall of PV was more vulnerable to ablation injury. The application of lower ablation parameters on the PV posterior wall would also lead to the IIR of the ablation point easily reaching the set threshold of 1.2, resulting in the area under the ROC curve was approaching to 0.5. This suggested that ablation parameters such as LSI were less accurate in determining ablation lesion existence at posterior wall of PV than at other PV segments. Moreover, CMR image resolution might also have an influence on this result. When ablation produced sufficient atrial tissue lesion but did not show high signal intensity on CMR image, the accuracy of ablation parameters in predicting atrial tissue lesion existence would also be less estimated.

Late gadolinium enhancement cardiac magnetic resonance has been used to visualize tissue lesion induced by radiofrequency ablation (15). Previous studies have defined ablation lesions as LGE sites with signal intensity 2 SD higher than healthy left atrial wall (23). IIR reflected the extent of myocardial tissue damage, and IIR ≤ 1.2 was the LGE-CMR threshold for healthy left atrial tissue in healthy individuals (13). Therefore, IIR > 1.2 identified the existence of left atrial tissue lesion. Ablation points were projected onto the post-processed left atrium LGE-CMR shells and IIRs of left atrial tissue corresponding to ablation points were obtained. We found that IIR increased with increasing LSI at non-posterior of PV, whereas the correlation was less marked at posterior of PV. The contrast agent entered into the cardiac tissue and presented high signal intensity when myocardial was damaged by radiofrequency ablation (24). The IIR value was mostly dependent on the amount of contrast agent distributed in the myocardium. At posterior wall of PV where the tissue lesion was relatively severe, the contrast agent distributed in the myocardial tissue might reach saturation. In this case, the IIR no longer continued to increase and reached a plateau at posterior wall of PV. Given the above results, we speculated that a real-time measurement of LSI during PVI might reflected the tissue lesion at non-posterior wall of PV in human bodies. Although the LSI has been well established, it is sometimes difficult to achieve first-pass PVI even with LSI-guided procedures (25). In our study, we found LSI was less accurate in determining acute ablation lesion at posterior wall of PV than at other areas under the ablation line. LSI is a multi-parametric index that considering contact force, power, and duration time of ablation. However, LSI does not take into account regional variations of left atrial tissue, such as composition and thickness. Our results suggested that LSI could be optimized to obtain a better novel parameter to reflect radiofrequency lesion, and LGE-CMR could be used as a tool to quantitative assess the predictive accuracy of optimized novel parameter to perform PVI in the future.

## 5. Study limitations

The present study has several limitations. First, this was a retrospective single center study, and selection bias could not be ruled out. Second, LGE-CMR examination could not be performed on patients with chronic kidney disease. Thus, the study results could not be generalized to such populations. Third, patients did not undergo LGE-CMR examination before radiofrequency ablation procedure. Therefore, we were unable to determine whether myocardial damage was entirely caused by radiofrequency ablation. There might had existed tissue damage of the left atrium under the ablation line. Fourth, patients did not undergo LGE-CMR re-examination 3 months after ablation procedure. Creating durable transmural radiofrequency lesions are quite important for the success rate of atrial fibrillation ablation, whether acute tissue lesion formation after PVI reflected by LGE was correlated with durable transmural radiofrequency lesion formation has not been explored in our study. Fifth, left atrial tissue damage was evaluated only in the acute phase, prospective studies with larger sample sizes and with long term re-examination of LGE-CMR are needed to identify the correlations between ablation characteristics and long-term clinical outcomes.

## 6. Conclusion

This study demonstrated that posterior of PV was more vulnerable to radiofrequency ablation lesion than non-posterior of PV when similar ablation parameters were applied. At posterior wall of PV, radiofrequency ablation needs to be more caution and does not require excessive ablation. LSI was a better predictor of tissue lesion existence following PVI than FTI, contact force, power, temperature, and duration time and there was a correlation between IIR and LSI at non-posterior wall of PV. Moreover, quantitative LGE-CMR IIR could be used as a tool to evaluate the relationship between ablation parameters and tissue lesion quality after PVI.

## Data availability statement

The original contributions presented in this study are included in the article/Supplementary material, further inquiries can be directed to the corresponding authors.

## Ethics statement

The studies involving human participants were reviewed and approved by the Institutional Ethics Committee of Tongji Hospital, Tongji Medical College, Huazhong University of Science and Technology (Approved ID: TJ-IRB20220814). Written informed consent for participation was not required for this study in accordance with the national legislation and the institutional requirements.

## Author contributions

LL, JL, QW, and BH designed the work. QW performed the study design, data collection, data analysis, and writing the manuscript. BH contributed to the data extraction, data collection, and quality assessment. SH, JG, and WS helped to interpret the data. TJ, DP, LM, YJ, LP, MZ, and SL helped to review and edit the manuscript. HL, DT, YL, CX, and XP helped the perform image post-processing. All authors approved the final manuscript.
