# Supplementary material for: Quantitative late gadolinium enhancement cardiac magnetic resonance analysis of the relationship between ablation parameter and left atrial tissue lesion following pulmonary vein isolation
Source: Front Cardiovasc Med. 2023 Jan 9;9:1030290. doi: 10.3389/fcvm.2022.1030290 (PMC9869251; doi:10.3389/fcvm.2022.1030290)
Supplement: Supplementary file 1 [file Image_1.PDF]

Supplementary Material

1. Supplementary Figure

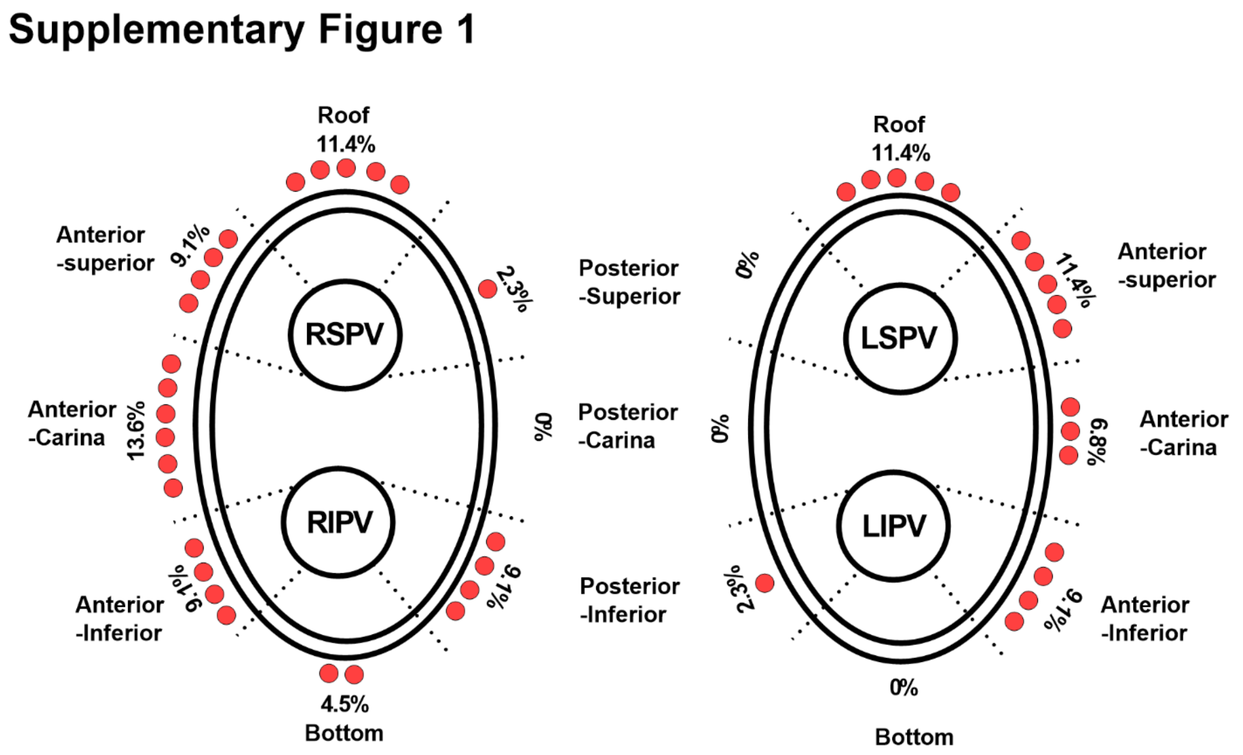

Supplementary Figure 1. Distribution of visual gaps around pulmonary vein segments. RSPV, right superior pulmonary vein, RIPV, right inferior pulmonary vein, LSPV, left superior pulmonary vein, LIPV, left inferior pulmonary vein.

Supplementary Figure 2

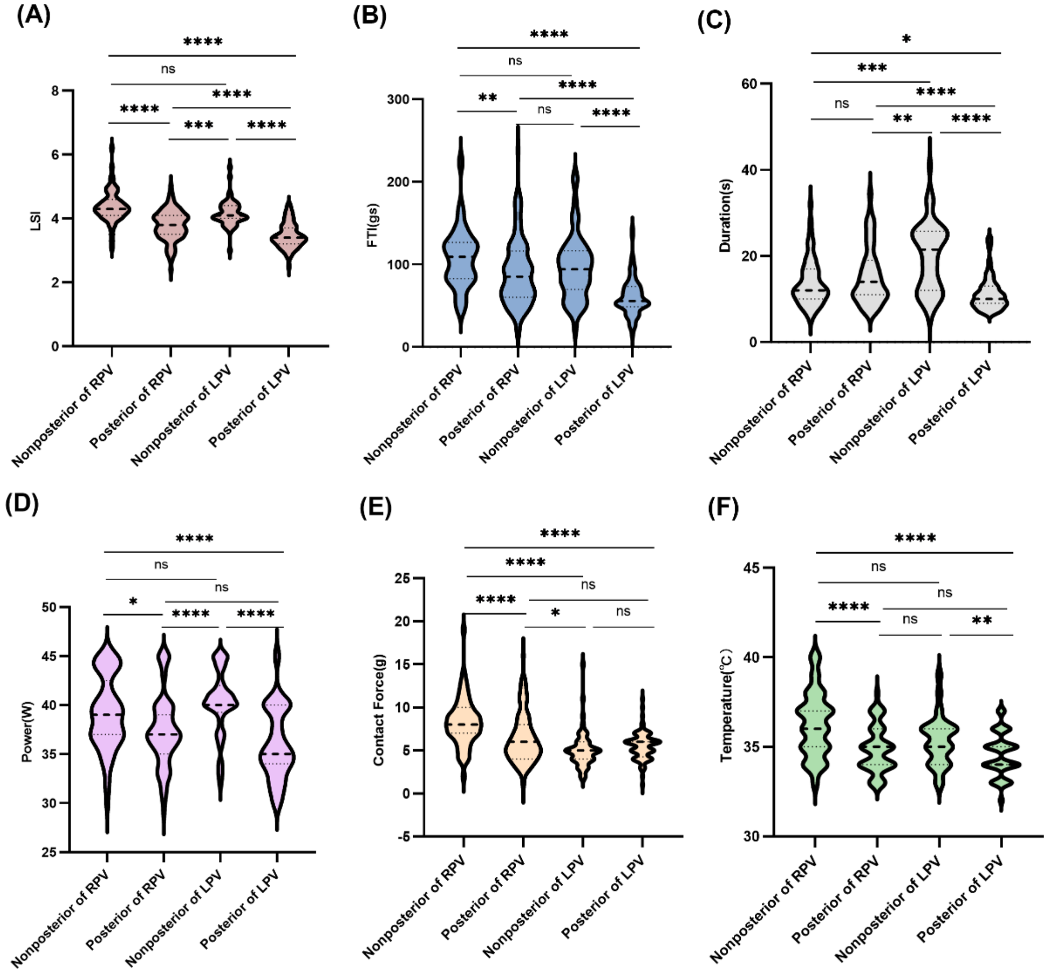

Supplementary Figure 2. Comparison of ablation parameters corresponding to tissue lesion points between different locations of left atrium. Comparison of (A) LSI, (B) FTI, (C) power, (D) duration, (E) contact force, (F) temperature applied during PVI corresponding to tissue lesion points between non-posterior of right PV, posterior of right PV, non-posterior of left PV and posterior of left PV. LSI, lesion size index, FTI, force–time integral, RPV, right pulmonary vein, LPV, left pulmonary vein.

Supplementary Figure 3

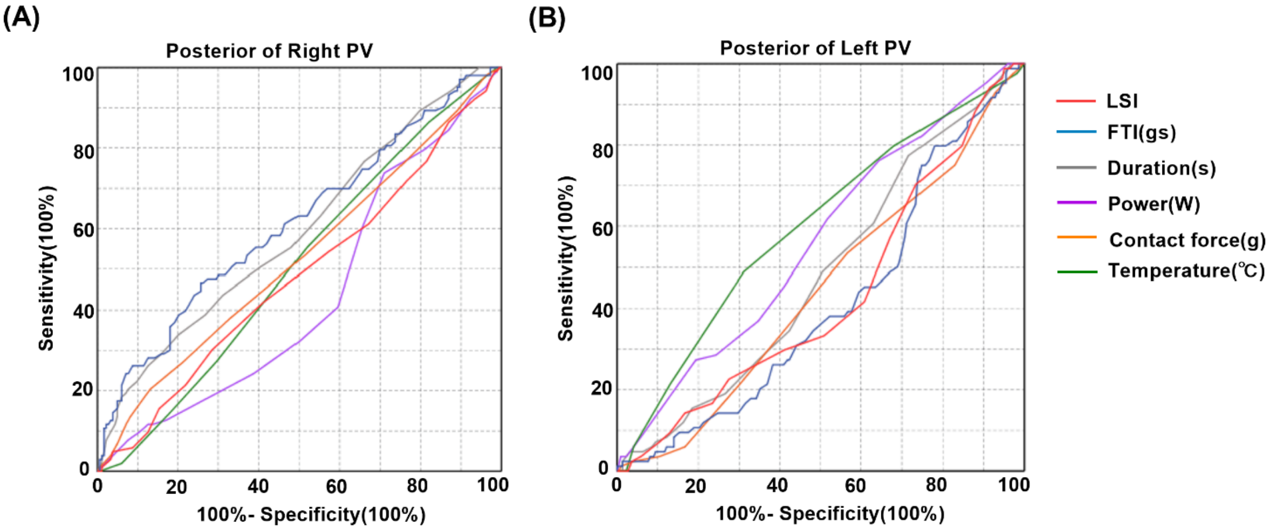

Supplementary Figure 3. Prediction of tissue lesion existence after pulmonary vein isolation at posterior of PV. (A) Receiver operating characteristic curve analysis for tissue damage existence predictability at posterior of right PV. Area under the ROC curve for LSI, FTI, contact force, temperature, power and duration was 0.484, 0.614, 0.528, 0.509, 0.433, and 0.594 respectively. (B) Receiver operating characteristic curve analysis for tissue damage existence predictability at posterior of left PV. Area under the ROC curve for LSI, FTI, contact force, temperature, power and duration was 0.431, 0.412, 0.444, 0.599, 0.561, and 0.483 respectively. LSI, lesion size index, FTI, force–time integral, PV, pulmonary vein.
